# Supplementary material for: Neural assemblies uncovered by generative modeling explain whole-brain activity statistics and reflect structural connectivity
Source: eLife. 2023 Jan 17;12:e83139. doi: 10.7554/eLife.83139 (PMC9940913; doi:10.7554/eLife.83139)
Supplement: Supplementary file 1. [file elife-83139-supp1.pdf]

Abbreviation	Full name	Abbreviation	Full name
th	Thalamus left	s	Subpallium left
ts	Torus semicircularis left	mos2	MO stripe 2 left
t	Tegmentum left	MOS2	MO stripe 2 right
mos1	MO stripe 1 left	vr	Vagal region left
TH	Thalamus right	OE	Olfactory epithelium right
TS	Torus semicircularis right	oe	Olfactory epithelium left
T	Tegmentum right	VR	Vagal region right
MOS1	MO stripe 1 right	IO	Inferior olive right
MOS5	MO stripe 5 right	nx	Vagus motor neurons left
mos4	MO stripe 4 left	NX	Vagus motor neurons right
MOS4	MO stripe 4 right	io	Inferior olive left
mos5	MO stripe 5 left	tl	Torus longitudinalis left
mon	Medial octavolateral nucleus left	TL	Torus longitudinalis right
MON	Medial octavolateral nucleus right	TeO	Tectum right
Ce	Cerebellum right	teo	Tectum left
ce	Cerebellum left	OB	Olfactory bulb right
MOS3	MO stripe 3 right	ob	Olfactory bulb left
mos3	MO stripe 3 left	ha	Habenula left
Pr	Pretectum right	Ha	Habenula right
pr	Pretectum left	irf	Intermediate reticular formation left
PT	Posterior tuberculum right	prf	Posterior reticular formation left
pt	Posterior tuberculum left	r	Raphe nucleus left
P	Pallium right	R	Raphe nucleus right
p	Pallium left	iRF	Intermediate reticular formation right
S	Subpallium right	pRF	Posterior reticular formation right
