# Supplementary material for: Neural assemblies uncovered by generative modeling explain whole-brain activity statistics and reflect structural connectivity
Source: eLife. 2023 Jan 17;12:e83139. doi: 10.7554/eLife.83139 (PMC9940913; doi:10.7554/eLife.83139)
Supplement: Supplementary file 2. — Abbreviations and example studies: Principal Component Analysis (PCA, Ahrens et al., 2012; Lopes-dos-Santos et al., 2013; Marques et al., 2020), Independent Component Analysis (ICA, Lopes-dos-Santos et al., 2013), k-means based algorithms (k-means, Panier et al., 2013; Chen et al., 2018; Stringer et al., 2019; Bartoszek et al., 2021), Non-Negative Matrix Factorization (NNMF, Mu et al., 2019), Variational Auto-Encoder (VAE, Tubiana et al., 2019b), Generalized Linear Model (GLM, Bishop, 2006), Boltzmann Machine (BM, Schneidman et al., 2006; Meshulam et al., 2017), compositional Restricted Boltzmann Machine (cRBM, this study). Question marks denote tasks that are in principle feasible but computationally expensive and/or not demonstrated. [file elife-83139-supp2.pdf]

Algorithm	PCA	ICA	k-means	NNMF	VAE	GLM	BM	cRBM
Learns probability distribution	No	No	No	No	Yes	No	Yes	Yes
Learns low-dimensional representation	Yes	Yes	No	Yes	Yes	No	No	Yes
Learns functional connectivity	No	No	No	No	?	Yes	Yes	Yes
Allows inhibiting connections	Yes	Yes	No	No	Yes	Yes	Yes	Yes
Scalable to large data sets	Yes	Yes	Yes	Yes	Yes	No	No	Yes
Perturbation prediction	No	No	No	No	?	No	Yes	Yes
Can infer assemblies	No	No	Yes	Yes	Yes	No	No	Yes
